# Design and Growth of P-Type AlGaN Graded Composition Superlattice

**DOI:** 10.3390/mi15121420

**Published:** 2024-11-26

**Authors:** Yang Liu, Xue Yang, Xiaowei Zhou, Peixian Li, Bo Yang, Zhuang Zhao, Yingru Xiang, Junchun Bai

**Affiliations:** 1 School of Advanced Materials and Nanotechnology, Xidian University, Xi’an 710071, China; yliu_333@stu.xidian.edu.cn (Y.L.);; 2 State Key Discipline Laboratory of Wide Band Gap Semiconductor Technology, Xidian University, Xi’an 710071, China

**Keywords:** superlattice, MOCVD, periodic thickness, hole concentration

## Abstract

A graded composition superlattice structure is proposed by combining simulation with experimentation. The structural factors affecting graded symmetric superlattices and graded asymmetric superlattices and their action modes are simulated and analyzed. A Mg-doped graded symmetric superlattice structure with high Al content, excellent structural quality, good surface morphology and excellent electrical properties was grown by MOCVD equipment. The Al_x_Ga_1−x_N superlattice with Al composition of 0.7 in the barrier exhibits a hole concentration of approximately 5 × 10^15^ cm^−3^ and a resistivity of 66 Ω·cm.

## 1. Introduction

Group III nitride semiconductor materials are also known as GaN-based semiconductor materials. They mainly include gallium nitride (GaN), aluminum nitride (AlN), indium nitride (InN) and their multicomponent alloys, such as ternary alloy aluminum gallium nitride (AlGaN), indium gallium nitride (InGaN), aluminum indium nitride (AlInN) and quaternary alloy aluminum indium gallium nitride (AlInGaN). Their band gap is continuously adjustable from the 6.2 eV of aluminum nitride (AlN) to the 3.4 eV of gallium nitride (GaN) and then to the 0.7 eV of indium nitride (InN), and a wide coverage range of a direct band gap from deep ultraviolet, to visible light, to the infrared region is realized [1,2,3,4]. Moreover, the nitride material can replace the Ga atom by doping the Si element or the Mg element to form donor or acceptor impurities, so as to realize effective N-type or P-type doping [5], which makes it possible to design and manufacture optoelectronic devices or electronic devices with specific functions, such as light-emitting diodes [6], lasers [7], photodetectors [8], and solar cells [9].

Compared with the first and second generation semiconductor materials, the third generation semiconductor materials face challenges such as having a high crystal defect density, an imperfect process, and a high production cost. Nevertheless, research on GaN-based light-emitting diodes (LEDs) and Laser Diodes (LDs) has achieved significant breakthroughs [10,11,12], driving substantial changes in the lighting industry. In fact, the outstanding performance of third generation semiconductor materials has not been fully tapped, and their development potential is huge, especially in the fields of deep ultraviolet photoelectric devices [13], power devices [14], electronic devices [15], and optical communication [16].

Al_x_Ga_1−x_N material has excellent growth controllability, which makes it an ideal choice for manufacturing energy-saving and environment-friendly deep ultraviolet electrical devices [13,17,18]. It is particularly noteworthy that nitride materials show extremely strong polarization characteristics, and their polarization field is measured in amounts as high as MV/cm [19]. A triangular potential well is generated in a structure such as an AlGaN/GaN heterojunction, and a two-dimensional electron gas (2DEG) is further formed [20], with a concentration as high as 10^13^ cm^−2^. At the same time, it is accompanied by superior properties such as a high saturated electron drift speed and a high breakdown electric field, which makes it show high application value in the field of high-power electronic devices [21,22]. Additionally, the conduction band off-set (CBO) of a nitride quantum structure can be continuously adjusted, and the CBO of an AlN/GaN system can reach 1.8 eV, thus realizing Inter-Sub-Band Transition (ISBT) infrared devices in optical an communication band [23].

An AlGaN ternary alloy material can continuously adjust the band gap in the range of 3.4~6.2 eV by adjusting the Al composition, and its corresponding light wavelength range is 200~360 nm, covering most areas from UVA to UVC, so it is considered as the first choice material for UV LED. An AlGaN-based ultraviolet LED shows many advantages, such as having no mercury pollution, an adjustable wavelength, a compact size, rapid response, high temperature resistance, radiation resistance, a low working voltage, and a long device life [13,24,25]. These advantages make it show great application potential in many fields such as lighting, disinfection, medical treatment, printing, storage, and communication. Theoretically, if you want to design a high-efficiency deep ultraviolet LED, you need to meet the following conditions. First of all, the crystal quality of the AlGaN epitaxial layer is high [26]. Secondly, a quantum well structure with high carrier confinement capability is needed [27]. And, what is most needed is an N-type electron injection layer and a P-type hole injection layer with high carrier injection efficiency [28,29]. Therefore, the improvement of photoelectric performance of a DUV LED is closely related to the above factors. There are still many challenges to realize a high-efficiency AlGaN-based deep ultraviolet LED, such as a low quantum efficiency [30], low current injection efficiency [31], and low light extraction efficiency [32]. Low quantum efficiency is the main factor limiting the development of an AlGaN-based ultraviolet LED, and one of the main factors limiting quantum efficiency is that it is difficult to achieve the efficient P-doping of AlGaN materials [5,29]. In the nitride material system, the technical difficulty of N-type doping is small, and the unintentionally doped nitride often presents a high N-type background carrier concentration, and the electron concentration of N-type AlGaN can easily reach 10^19^ cm^−3^, while the P-type doping of nitride is more difficult. The main factors limiting the P-type doping of nitrides include the low solubility of Mg atoms, high activation energy, and impurity compensation [33,34].

In order to solve the challenge of P-type doping in AlGaN, many strategies have been explored. These methods mainly start with inhibiting the defect of the self-compensation process, improving the solubility of Mg, and reducing the activation energy of Mg in AlGaN. These methods include uniform doping [35,36], δ doping, modulation doping [37], SL (superlattice) doping [38], polarization-induced doping [39], and three-dimensional doping [40], all of which have a positive impact on the P-type doping of AlGaN materials. However, these research schemes are simple in the way of performance regulation, and the doping efficiency is improved only by using a single structure in the P-type AlGaN layer, which often ignores the influence of the synergistic effect of multiple structures on the energy band structure and electrical properties. It is necessary to explain the electrical phenomena caused by the synergistic effect of the two doping methods by combining growth with simulation technology.

In this work, a graded Al superlattice structure of a potential well layer is proposed, and the effectiveness of the graded Al superlattice is verified by the simulation of a traditional superlattice structure and a graded Al superlattice structure. The non-graded superlattice structure and the graded superlattice structure were grown by MOCVD epitaxy. In addition, an ultraviolet–visible photometer (UV-VIS) is used to analyze the transmittance of the AlGaN superlattice. High-resolution X-ray diffraction (HR-XRD) is used to characterize the superlattice structure. An atomic force microscope (AFM) is used to analyze the surface of the superlattice structure. In addition, the Hall effect test has been used to analyze the resistivity of the AlGaN superlattice, which can predict the doping efficiency of a superlattice structure.

## 2. Simulation of Traditional Superlattice and Graded Superlattice

**Figure 1:**
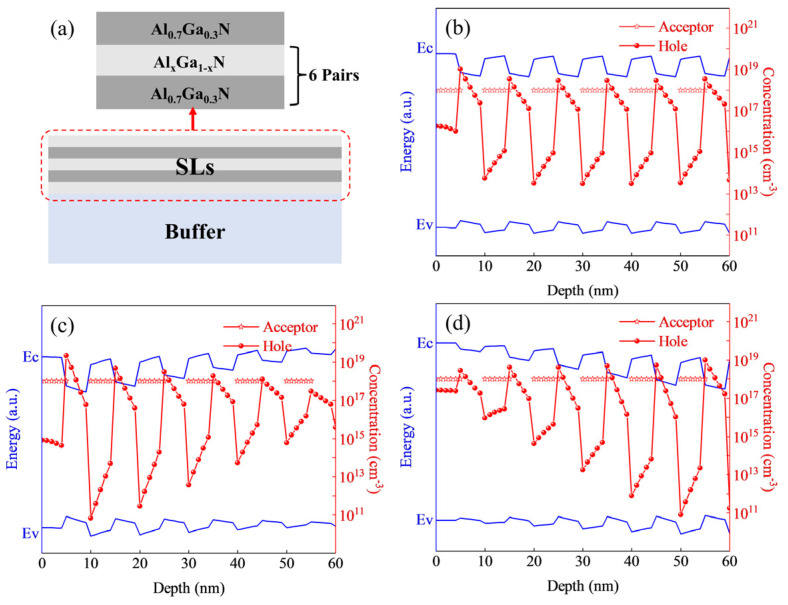
(**a**) Superlattice structure model; band diagram and hole density distribution of the superlattice structure: (**b**) traditional superlattice structure; (**c**) Al composition decreasing along the c-axis; (**d**) Al composition increasing along the c-axis.

In this work, the Silvaco TCAD software (version 2014) was employed to simulate AlGaN superlattice structures, exploring the effects of varying P-type doping concentrations, superlattice configurations, periodicity, and buffer layer designs. A structural diagram of the design is presented in Figure 1a.

All the simulated structures use 1.5 μm of Al_0.5_Ga_0.5_N as a buffer, and six periodic superlattice structures are grown on it. The structural schematic diagram is shown in Figure 1a. The hole concentration and energy band structure of the AlGaN superlattice are simulated by adjusting the Al component X of the barrier layer. The Al composition of the barrier layer of all samples is set to 0.7, and the thickness of the barrier layer and the potential well layer is 5 nm. Sample A adopts a superlattice with a traditional structure, and the Al composition of the potential well layer is x = 0.35 (Al_0.35_Ga_0.65_N/Al_0.7_Ga_0.3_N). Sample B adopts a superlattice structure with a graded Al composition in its potential well. Along the c-axis of the buffer layer, the Al composition (x) in the potential well region gradually decreases from 0.6 to 0.1, with a change of 0.1/period. Sample C also adopts the superlattice structure with a graded Al composition in the potential well. In contrast to sample B, the Al composition (x) in the potential well region of sample C increases from 0.1 to 0.6 along the c-axis of the buffer layer, with a change of 0.1/period. In our previous study, it was found that doping in the barrier region is beneficial to improve the efficiency of P-doping, so we chose to only carry out P-type doping in the barrier region with a doping concentration of 1 × 10^18^ cm^−3^. These parameters are set to ensure that the average Al composition in the superlattice region, that is, that in the P-type region, is 0.35 and that the average doping concentration is 5 × 10^17^ cm^−3^, to simulate the hole concentration and energy band structure, and to explore the influence mechanism of the hole concentration in the superlattice region.

Figure 1a shows the simulation structure model. As depicted in Figure 1b, the energy band structure of the traditional superlattice exhibits a uniform zigzag pattern, with an average hole concentration of 7.17 × 10^17^ cm^−3^ in the superlattice region. In contrast, the oscillation of the energy band structure in the graded Al-composition superlattice shown in Figure 1c,d increases in the direction where the Al composition of the quantum well decreases. Figure 1c, where the Al composition of the well decreases along the c-axis, shows an average hole concentration of 7.65 × 10^17^ cm^−3^ in the superlattice region, while Figure 1d, with an increasing Al composition along the c-axis, shows an average hole concentration of 7.71 × 10^17^ cm^−3^. From Figure 1b,c, it is clear that the peak in hole concentration consistently appears at the junctions between the barriers and wells. As previously studied, under the strong polarization field of the AlGaN material in the superlattice structure, band bending occurs at the superlattice interfaces. This causes the acceptor levels in the barrier layer to activate and shift towards the valence band maximum in the nearby well region, facilitating the easier ionization of acceptors in the barrier layer and thereby generating holes. Consequently, this effect significantly enhances the hole concentration. Moreover, graded Al-composition superlattice structures can provide a higher hole concentration. This is due to the strong polarization field generated by the linear variation in Al composition along the well layers. The polarization field, resulting from both spontaneous polarization and the piezoelectric polarization induced by the stress from the buffer layer, induces the ionization of acceptors at each interface. This polarization-induced hole generation further contributes to the higher hole concentration, underscoring the advantage of graded Al-composition superlattice structures in enhancing P-type doping.

**Figure 2:**
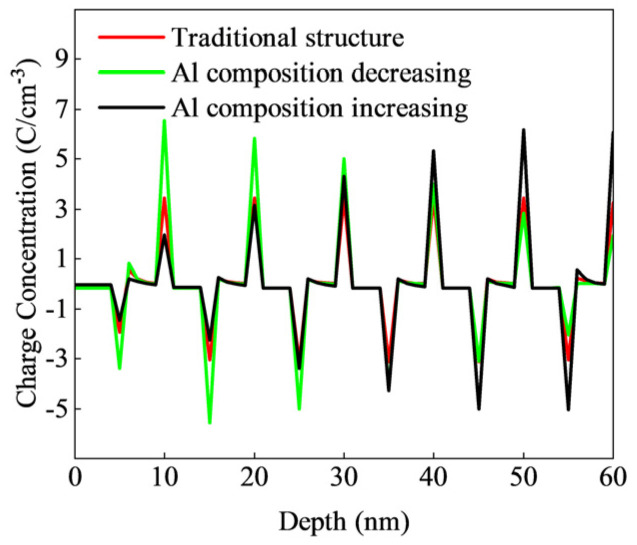
Charge density distribution of different superlattice structures.

The variation in interface charge, caused by interface state density and internal lattice strain, is a critical factor influencing the P-type doping efficiency of the superlattice structure. Lattice mismatch, arising from differing Al compositions between the superlattice well and the barrier layer, leads to changes in the interface charge, as illustrated in Figure 2. The red, green, and black solid lines in Figure 2 represent the charge densities for the three superlattice structures shown in Figure 1b–d, respectively. It can be observed that the positive charge at the interface weakens, while the negative charge enhances the hole concentration. The interface charge and the activation mechanism of Mg within the superlattice collectively influence the hole concentration in the superlattice region.

When the difference in Al composition between the well and barrier regions is small, the local hole concentration at the well/barrier interface decreases. This phenomenon is illustrated in Figure 1, where the hole concentration in the potential well region decreases while the concentration in the barrier region increases. The primary cause of the reduced hole concentration in the potential well region is the tendency of Mg doped in the barrier layer to capture electrons at the top of the valence band in the potential well, becoming ionized and leaving holes in the well. As the Al composition in the potential well region increases, the activation energy of the Mg acceptor in the barrier layer also increases, making it more difficult for Mg to ionize and thereby reducing the hole concentration in the well. The increase in hole concentration in the barrier region is primarily due to a decrease in the positive charge concentration at the interface as the Al composition of the well and barrier approach similarity (at 10, 20, 30, 40, 50, and 60 nm in Figure 2). This reduction in positive charge concentration enhances the hole concentration in the barrier region.

**Figure 3:**
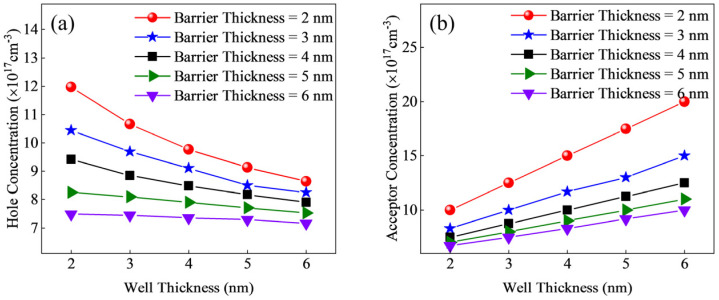
Different well/barrier thicknesses: (**a**) hole concentration; (**b**) doping concentration.

We redesigned the structure of sample C, which exhibited the highest hole concentration in Figure 1d, and simulated superlattice structures with varying barrier thicknesses. As shown in Figure 3a, we modified the thickness of the barrier and well layers. To ensure an average doping concentration of 5 × 10^17^ cm^−3^ in the superlattice region, the doping concentration in the barrier region was adjusted, as presented in Figure 3b. The corresponding hole concentration results are shown in Figure 3a. From the figure, it is evident that reducing the well and barrier thicknesses contributes to an increase in hole concentration. Moreover, the change in barrier width has a greater impact on hole concentration than the change in well width. This is because doping occurs in the barrier region, and to maintain a consistent average doping concentration in the superlattice, the doping concentration in the well region is adjusted. This variation in doping concentration strongly affects the ionizable acceptor levels near the well–barrier interfaces, thus influencing the hole concentration.

**Figure 4:**
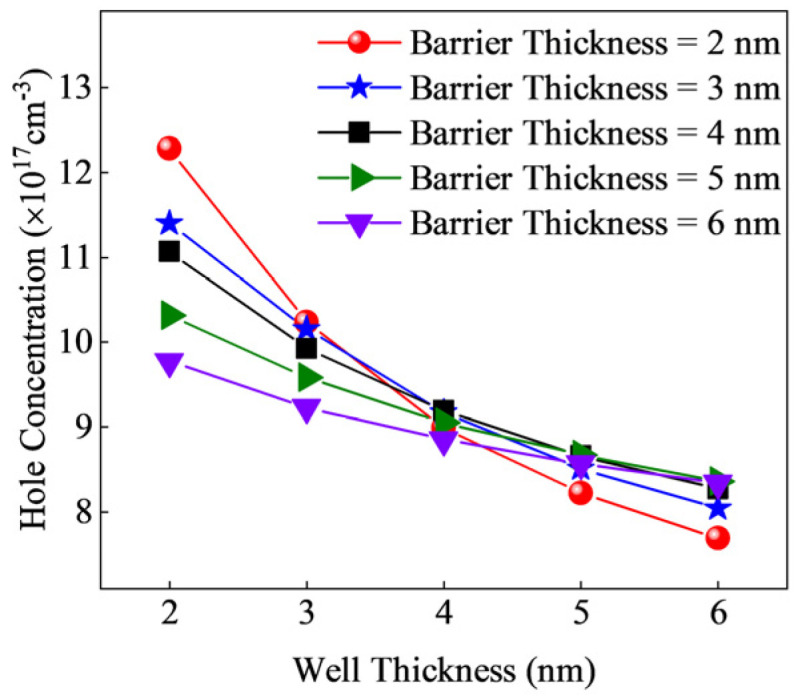
Hole concentration with uniform doping in the well and barrier regions.

To eliminate the influence of the acceptor doping concentration in the barrier region, we applied P-type doping to both the well and barrier regions, with a doping concentration of 1 × 10^18^ cm^−3^. The resulting hole concentrations are shown in Figure 4. Across all structures, a higher hole concentration is achieved with smaller periodic thicknesses. As the well thickness increases, the rate of decrease in the hole concentration slows for all structures. This is because the increased well thickness leads to a flattening of the band oscillations at the well–barrier interfaces, which hinders acceptor ionization.

## 3. Experiment

All samples were grown on sapphire substrates using a metal–organic chemical vapor deposition (MOCVD) system (AIXTRON CRIUS 2, Herzogenrath, Germany). The buffer layers, from bottom to top, consist of 500 nm of AlN, 500 nm of Al_0.75_Ga_0.25_N, and 1.5 μm of Al_0.5_Ga_0.5_N. Trimethylaluminum (TMAl), trimethylgallium (TMGa), and ammonia (NH_3_) were employed as precursors, with hydrogen (H_2_) serving as the carrier gas. P-type doping was performed exclusively in the superlattice barrier layers, using Cp_2_Mg as the dopant. The superlattice structure was maintained at 6 periods.

The detailed growth procedure for Mg-doped P-type AlGaN epitaxial structures is as follows:

(1) Prior to epitaxial growth, the c-plane sapphire substrate is placed on a graphite susceptor inside the reaction chamber, and the susceptor is heated to 1100 °C to remove contaminants from the sapphire surface;
(2) While maintaining the susceptor at 1100 °C, NH_3_ is introduced into the reaction chamber to perform nitridation on the sapphire substrate;
(3) Subsequently, TMAl, TMGa, and NH_3_ are sequentially introduced into the chamber to grow epitaxial layers of 500 nm of AlN, 500 nm of Al_0.75_Ga_0.25_N, and 1.5 μm of Al_0.5_Ga_0.5_N on the sapphire substrate;
(4) Following this, an Mg-doped P-type Al_x_Ga_1−x_N/Al_y_Ga_1−y_N superlattice is grown on the Al_0.5_Ga_0.5_N buffer layer, with the well and barrier layer thicknesses controlled by growth time, resulting in a superlattice structure with 6 periods. P-type modulation doping is applied, where only the barrier layer is doped with Cp_2_Mg at a flow rate of 220 sccm, while the well layer remains undoped;
(5) Finally, the grown samples are annealed in a nitrogen atmosphere to activate the Mg acceptors.

To evaluate the effectiveness of the graded Al composition superlattice compared to the conventional superlattice structure, two Mg-doped P-type AlGaN epitaxial structures, A1 and A2, were fabricated. Both samples have a barrier layer Al composition of 0.1, with the only difference being the Al composition in the AlGaN superlattice well. For sample A1, the well Al composition is x = 0.35, whereas for sample A2, x gradually decreases from 0.6 to 0.1.

**Figure 5:**
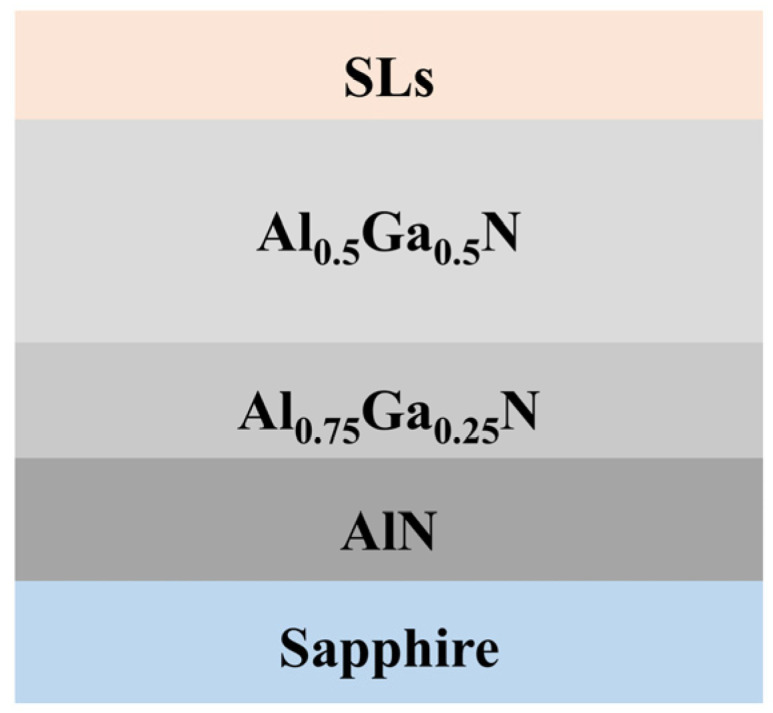
Information of superlattice structure.

To verify the effectiveness of Al composition-graded superlattices with different periodic thicknesses, samples B1 and B2 were prepared. The barrier layer in sample B1 has a thickness of 2 nm, while the well layer is 5 nm thick. In sample B2, the barrier layer also has a thickness of 2 nm, but the well layer thickness is reduced to 3 nm. Detailed information about the samples is shown in Table 1. The sample information is presented in Figure 5.

**Figure 6:**
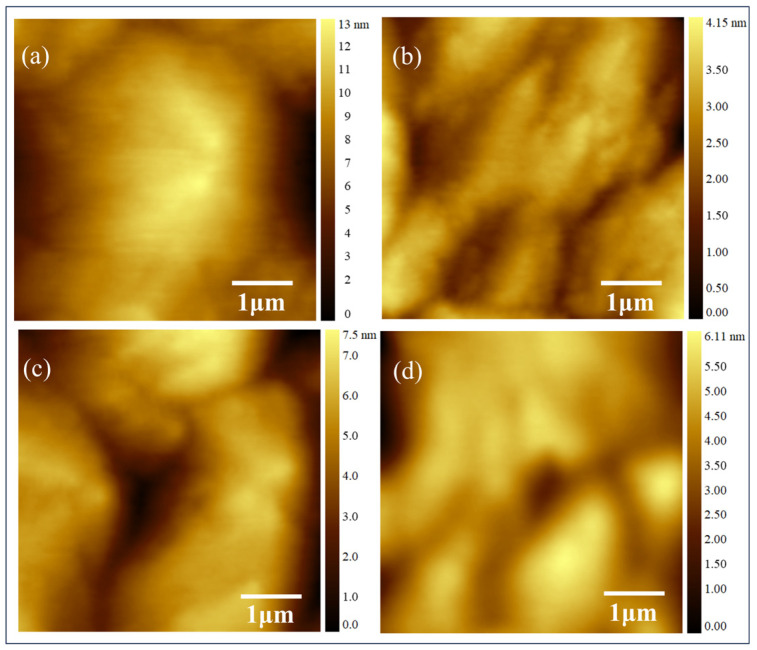
AFM image: (**a**) sample A1; (**b**) sample A2; (**c**) sample B1; (**d**) sample B2.

The surface morphology of samples A1 and A2 was characterized using an AFM, as shown in Figure 6. The surface roughness root mean square (RMS) value of sample A1, which utilizes a traditional superlattice structure, is 2.4 nm. For sample A2, which uses a graded superlattice structure, the RMS value is reduced to 1.49 nm. Distinct small island-like protrusions can be observed on the surface of sample A1. In contrast, these protrusions are significantly reduced in sample A2, resulting in a smoother surface. It is evident that the surface roughness is significantly reduced when a graded composition superlattice is employed. This improvement in surface morphology can be attributed to the graded Al composition in the superlattice, which effectively releases residual internal stress in the Al_x_Ga_1-x_N/Al_0.7_Ga_0.3_N superlattice structure. Sample B1 has a barrier thickness of 5 nm/2 nm, while sample B2 has a barrier thickness of 3 nm/2 nm. The root mean square (RMS) roughness values for samples B1 and B2 are 1.75 nm and 1.46 nm, respectively. Sample B2 exhibits the lowest surface roughness, indicating a superior surface morphology. Samples B1 and B2 follow the same growth mode, while samples A1 and A2 do not exhibit the same step-flow growth mode as these two samples. The observed phenomenon can be attributed to the greater thickness of sample A2, which leads to a higher defect density during the growth process, and results in a larger surface roughness for the sample.

**Figure 7:**
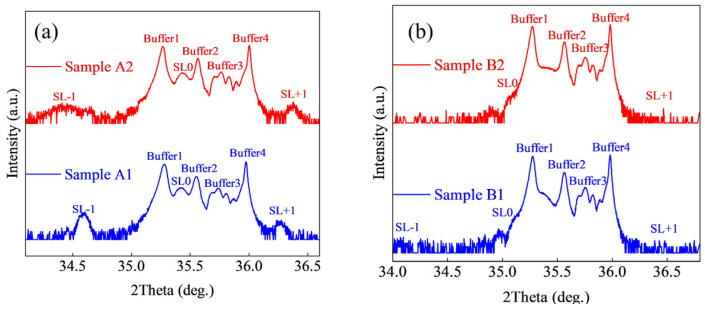
HR−XRD (0002) 2theta−omega scanning fitting curves of (**a**) sample A1 and A2, and (**b**) sample B1 a nd B2.

Figure 7a presents the HR-XRD 2θ-ω scanning curves for samples A1 and A2. The scanning curves for samples A1 and A2 clearly exhibit multiple diffraction peaks. Notable diffraction peaks are observed at 35.27° and 35.98°. The peak at 35.27° corresponds to the Al_0.5_Ga_0.5_N layer, while the peak at 35.98° corresponds to the AlN buffer layer. Given that the superlattice structure consists of a multilayer film, its diffraction pattern displays a prominent Bragg diffraction peak surrounded by a series of satellite peaks. The intensity and positional relationships of the satellite peaks can be utilized to characterize the thickness, compositional variations, and stress within the multilayer film, as previously described in our research [41]. The positions of the zero-order and higher-order peaks of the superlattice were fitted from the diffraction pattern, with the results presented in Table 2. The fitted peak positions of the superlattice enable the estimation of the periodic thickness, which was calculated to be consistent with the target periodic thickness of 10 nm.

As shown in Figure 7b, the periodic thicknesses of samples B1 and B2 were estimated based on the spacing between the zero-order peak and other higher-order peaks, with sample B1 having a periodic thickness of 7.3 nm and sample B2 having a periodic thickness of 4.9 nm. These calculated results align with the target periodic thicknesses of 7 nm for sample B1 and 5 nm for sample B2. Furthermore, the strain (average lattice mismatch rate) between the superlattice structure and the substrate was estimated based on the substrate diffraction peak position and the zero-order peak position. The calculated results indicate that the strain for sample A1 is −0.87%, that for sample B1 is −0.714%, and for sample B2, it is −0.765%. It is evident that among the samples with different well and barrier thicknesses, sample B1 exhibits the lowest strain value, indicating the lowest average lattice mismatch rate and the best crystallinity. The reduction in strain values during the process of shortening the barrier while keeping the well thickness fixed demonstrates an improvement in crystallinity. Conversely, during the process of decreasing the well thickness while keeping the barrier thickness fixed, an increase in strain values indicates a deterioration in crystallinity. Therefore, it can be concluded that the crystallinity of the samples improves during the processes of increasing the well thickness or reducing the barrier thickness.

**Figure 8:**
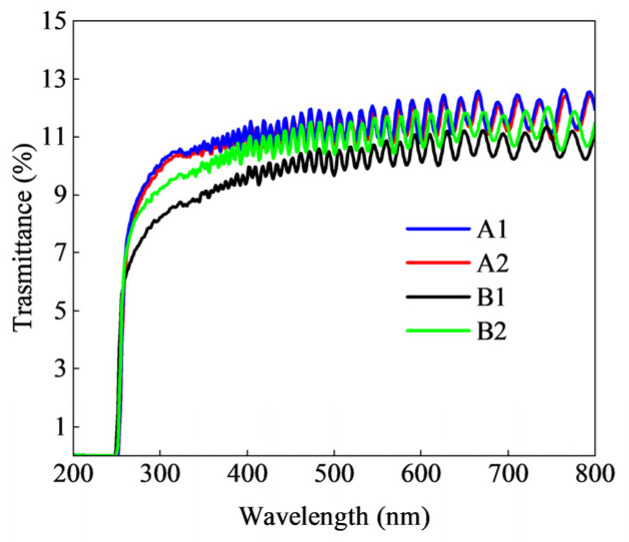
UV-VIS transmittance curves of samples.

Figure 8 presents the ultraviolet transmission spectra for all samples. The transmission curves clearly indicate that the transmission rate of AlGaN superlattice materials ranges from 10% to 12% within the wavelength range of 320 nm to 800 nm, with observable periodic interference fringes. At wavelengths below 260 nm, the transmission rates for all samples rapidly decline to zero. As the wavelength gradually decreases, the photon absorption capacity of AlGaN materials continues to increase. In the range of 200 nm to 250 nm, the transmission rate approaches zero, indicating that AlGaN materials have reached their absorption limit and begin to participate in the short-wave ultraviolet absorption process. All samples demonstrate favorable ultraviolet transmission rates, indicating that the designed gradient composition superlattice structure exhibits excellent ultraviolet transmission performance, meeting the design specifications.

From Figure 8, it can be observed that all samples exhibit oscillating curves in their transmission rates, indicating a low longitudinal compositional gradient, with relatively smooth interfaces and surfaces, thereby demonstrating the high quality of the samples. Numerous studies indicate that the steepness of the absorption edge in transmission spectra reflects the internal quality of the material. When defects are present in the samples, the transmission spectra can become distorted, leading to the appearance of reflection peaks, where a certain intensity of light is scattered away instead of entering the substrate effect region, thereby affecting its optical performance. A steep absorption edge indicates a smaller extinction coefficient, which is indicative of higher crystal quality; conversely, when the slope of the absorption edge becomes gentler, the extinction coefficient increases, leading to the significant consumption of incident light at the interface and consequently reducing the quality of the crystal. Figure 8 clearly shows that samples A1 and A2 exhibit the highest steepness, followed by B2, while sample B1 ranks the lowest. Additionally, it was found that as the thickness of the samples increases, the oscillation amplitude of the transmission spectra gradually decreases. Sample A1 has the shortest oscillation period, while the oscillation fluctuations of sample B1 are slightly greater than those of A1 and A2, with sample B2 exhibiting the largest oscillation amplitude. Therefore, it can be inferred that the thicknesses of the different samples are such that A1 and A2 are of a similar thickness and the thickest, B1 is in the middle, and B2 is the thinnest, which is consistent with the actual conditions of the samples.

**Figure 9:**
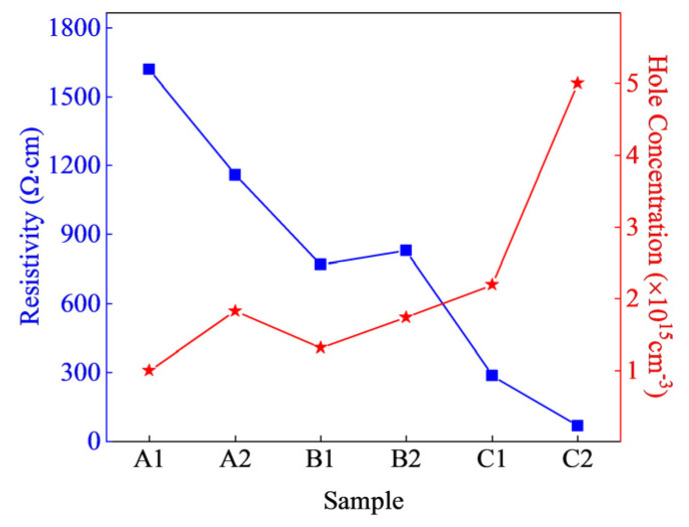
Resistivity and hole concentration of all samples.

To further investigate the electrical properties of different superlattice structure samples, contact Hall measurements were conducted using indium electrodes. The electrical properties of the different samples were characterized and analyzed, with the results presented in Figure 9. According to the test results, the electrical performance of sample A2, which utilizes a gradient composition superlattice structure, is superior to that of sample A1, which employs a non-gradient superlattice structure. A comparison of the hole concentrations among the different samples reveals that sample A2 exhibits a higher hole concentration value of 1.82 × 10^15^ cm^−3^ under the same average Al composition, period thickness, doping concentration, and doping method. This is because, when a superlattice structure is employed, the holes generated by the barriers can transfer into the wells, resulting in a significant accumulation of two-dimensional hole gas (2DHG), thereby increasing the average hole concentration. Consequently, the hole concentrations of both samples A1 and A2 reach the order of 10^1^⁵ cm⁻^3^. However, sample A2 utilizes a gradient composition superlattice structure, where the varying Al composition generates a strong polarization field within the superlattice. This robust polarization field, resulting from both piezoelectric and spontaneous polarization, facilitates the ionization of the Mg dopants, thereby providing a greater number of holes. In summary, the electrical performance of the gradient composition superlattice is superior.

The comparison of the resistivity among different samples, as shown in the results from Figure 9, indicates that sample B1, with a well thickness of 5 nm and a barrier thickness of 2 nm, exhibits the lowest resistivity of 769 Ω·cm, suggesting its superior electrical performance. Conversely, sample A1, with a well thickness of 5 nm and a barrier thickness of 5 nm, displays the highest resistivity at 1617 Ω·cm, which is approximately 50% higher than that of sample B1. Sample B2, with a well thickness of 3 nm and a barrier thickness of 2 nm, has a resistivity of 829 Ω·cm. In the previous simulation, as shown in Figure 1, it was found that improving hole concentration is facilitated by maintaining a uniform average Al composition in the superlattice region and adopting a gradient Al composition in the potential well layer. In the comparison of resistivity between the samples, the order is B1 < B2 < A2 < A1, indicating that the graded Al composition superlattice structure enhances P-type doping efficiency more effectively than the traditional superlattice structure. Comparing the resistivity of samples A1 and A2 reveals that the Al composition gradient in the superlattice potential well region improves P-type doping efficiency. Furthermore, comparing the resistivity of samples A2 and B1 shows a significant reduction in resistivity when the well thickness remains constant while the barrier thickness is reduced. Conversely, when the barrier thickness is kept constant and the well thickness is reduced, as in the comparison between samples B1 and B2, the resistivity of sample B2 actually increases, which is unfavorable for enhancing hole concentration. These comparisons clearly demonstrate that increasing the well thickness or decreasing the barrier thickness benefits the P-type doping effectiveness of the gradient asymmetric superlattice structure, which is consistent with the simulation results.

As shown in Figure 9, we found that sample B1 shows the lowest resistivity. Therefore, we adopted the structure of sample B1 to improve the flow rate of Cp_2_Mg. When growing a superlattice structure, the flux and the time of the fixed metal source and the N source can grow the same superlattice as the B1 structure, the flow rate of Cp_2_Mg is increased, and the growth time is unchanged, which is beneficial to more Mg entering the lattice position and increasing the doping concentration. The purpose of this is to increase the concentration of the Mg acceptor to be as much as possible to obtain a higher hole concentration. 

**Figure 10:**
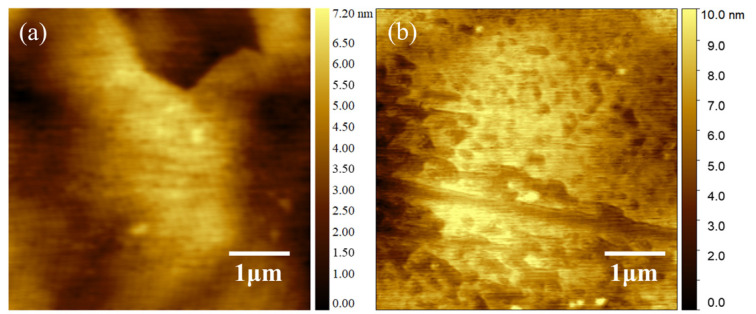
AFM images: (**a**) sample C1; (**b**) sample C2.

The flow rate of Cp_2_Mg in the barrier region was increased to 280 sccm and 320 sccm, resulting in the growth of samples C1 and C2. The surface morphology of samples C1 and C2 is illustrated in Figure 10, where the root mean square (RMS) roughness values are 1.46 nm and 2.86 nm, respectively. As shown in Figure 10, sample C1 continues to maintain a step-flow growth mode. In contrast, the surface of sample C2 is rough and lacks a flat appearance. This roughness can be attributed to the increased flow rate of Mg, which leads to a higher accumulation of defects during the growth process, resulting in a rougher surface texture. When the flow rate of Cp_2_Mg reached 320 sccm, the highest hole concentration of approximately 5 × 10^15^ cm^−3^ was obtained, with a corresponding resistivity of 66 Ω·cm, as shown in Figure 9.

**Figure 11:**
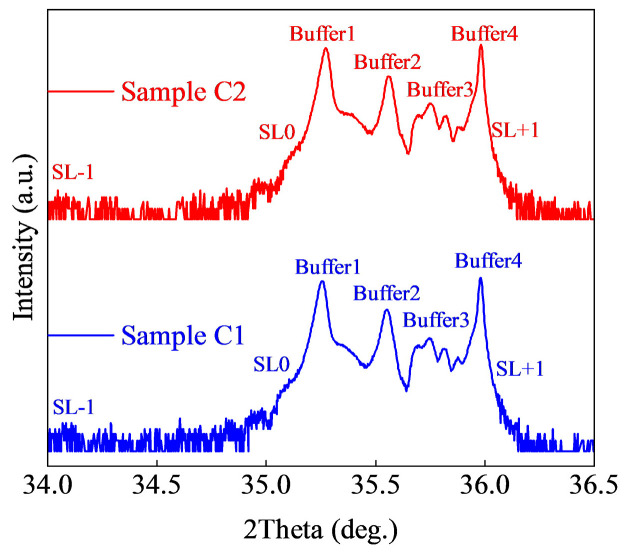
HR-XRD (0002) 2theta-omega diffraction of samples C1 and C2.

Figure 11 shows the HR-XRD diffraction patterns of samples C1 and C2. Two diffraction patterns that are similar to that of sample B1 in Figure 7b can be seen. The information of superlattice peak position is shown in Table 2.

## 4. Conclusions

This study combines simulation and epitaxial growth to investigate the superior doping effects of gradient composition superlattices, and to explore the mechanisms underlying both gradient symmetric and gradient asymmetric superlattices in detail. The results indicate that the gradient composition asymmetric superlattice structure with a varying Al component in the potential well can provide a higher hole concentration, and that reducing the period thickness effectively enhances the hole concentration. This is attributed to the significant influence of polarization effects on the energy band structure oscillation amplitude and period within the gradient composition asymmetric superlattice structure with a varying Al component, which strongly affects hole generation.

## Figures and Tables

**Table 1:** Information on wells and barriers of superlattice structures.

Sample		A1	A2	B1	B2
Well	Al Composition	0.35	x	x	x
	Thickness (nm)	5	5	5	3
Barrier	Al Composition	0.7	0.7	0.7	0.7
	Thickness (nm)	5	5	2	2
	Cp_2_Mg Flux (sccm)	220	220	220	220

**Table 2:** HR−XRD peak position and structural information.

Sample	A1	A2	B1	B2	C1	C2
SLs(−1)	34.59°	34.43°	34.13°	34.47°	34.12°	34.1°
SLs(0)	35.43°	35.39°	35.09°	35.25°	35.1°	35.1°
SLs(+1)	36.27	36.35°	36.05°	36.38°	36.05°	36.05°
Buffer 1	35.27°	35.27°	35.27°	35.27°	35.27°	35.27°
Buffer 2	35.72°	35.74°	35.73°	35.72°	35.73°	35.72°
AlN	35.98°	35.98°	35.98°	35.98°	35.98°	35.98°
SLs Al Composition	0.53	0.53	0.45	0.49	0.45	0.45
SLs Periodic Thickness (nm)	10.6	9.6	7.3	4.9	7.3	7.4

## Data Availability

All data that support the findings of this study are included within the article.
